# Pelvic lymph node mapping in prostate cancer: examining the impact of PSMA PET/CT on radiotherapy decision-making in patients with node-positive disease

**DOI:** 10.1186/s40644-024-00742-3

**Published:** 2024-07-29

**Authors:** Ben Furman, Tal Falick Michaeli, Robert Den, Simona Ben Haim, Aron Popovtzer, Marc Wygoda, Philip Blumenfeld

**Affiliations:** 1 Department of Radiation Oncology, Sharett Institute of Oncology, Hadassah Medical Center, Faculty of Medicine, Hebrew University of Jerusalem, POB 12272 Jerusalem, 9112002 Israel; 2 Department of Radiation Oncology, Thomas Jefferson, Philadelphia, PA USA; 3 Department of Nuclear Medicine and Medical Biophysics, Faculty of Medicine, Hebrew University of Jerusalem, Hadassah Medical Center, Jerusalem, Israel; 4 Department of Medical Oncology, Sharett Institute of Oncology, Hadassah Medical Center and Faculty of Medicine, Hebrew University of Jerusalem, Jerusalem, Israel

## Abstract

**Introduction:**

Prostate Specific Membrane Antigen (PSMA) imaging with Positron Emission Tomography (PET) plays a crucial role in prostate cancer management. However, there is a lack of comprehensive data on how PSMA PET/CT (Computed Tomography) influences radiotherapeutic decisions, particularly in node-positive prostate cancer cases. This study aims to address this gap by evaluating two primary objectives: (1) Mapping the regional and non-regional lymph nodes (LNs) up to the aortic bifurcation and their distribution using conventional methods with CT compared to PSMA PET/CT, and (2) assessing the impact of PSMA PET/CT findings on radiotherapeutic decisions.

**Methods:**

A retrospective analysis of 95 node-positive prostate cancer patients who underwent both CT and PSMA PET/CT imaging prior to primary radiotherapy and androgen deprivation therapy (ADT) was conducted. The analysis focused on identifying LNs in various regions including the common iliac, external iliac, internal iliac, obturator, presacral, mesorectal, inguinal, and other stations. Treatment plans were reviewed for modifications based on PSMA PET/CT findings, and statistical analysis was performed to identify predictors for exclusive nodal positivity on PSMA PET/CT scans.

**Results:**

PSMA PET/CT identified additional positive nodes in 48% of cases, resulting in a staging shift from N0 to N1 in 29% of patients. The most frequent metastatic LNs were located in the external iliac (76 LNs; 34%), internal iliac (43 LNs; 19%), and common iliac (35 LNs; 15%) stations. In patients with nodes only detected on PSMA PET the most common nodes were in the external iliac (27, 40%), internal iliac (13, 19%), obturator (11, 15%) stations. Within the subgroup of 28 patients exclusively demonstrating PSMA PET-detected nodes, changes in radiotherapy treatment fields were implemented in 5 cases (18%), and a dose boost was applied for 23 patients (83%). However, no discernible predictors for exclusive nodal positivity on PSMA PET/CT scans emerged from the analysis.

**Discussion:**

The study underscores the pivotal role of PSMA PET/CT compared to CT alone in accurately staging node-positive prostate cancer and guiding personalized radiotherapy strategies. The routine integration of PSMA PET/CT into diagnostic protocols is advocated to optimize treatment precision and improve patient outcomes.

## Introduction

Prostate cancer stands as the most prevalent malignancy among men and ranks third in terms of mortality [1]. A pivotal focus in recent advancements involves Prostate Specific Membrane Antigen (PSMA), a transmembranal protein unique to prostate epithelial cells whose overexpression in tumor cells correlates with cancer progression [2]. Positron Emission Tomography (PET) PSMA utilizes a marked ligand to track PSMA expression in the body, aiding in the identification of lymphatic and metastatic spread, crucial for tumor staging [2]. Moreover, it has been demonstrated to have significantly higher detection rates compared to other PET-tracers [3] and more likely to detect cancer in the recurrent setting [4].

In presumed localized high risk prostate cancer, PSMA PET/CT has also prospectively demonstrated improved sensitivity and specificity compared to conventional imaging with a higher detection rate of nodal and metastatic lesions thereby resulting in significant treatment management change [5]. While approximately 12% of prostate cancer patients exhibit positive lymph nodes (LNs) at diagnosis using conventional staging methods [6], this is expected to rise with the advent of PSMA PET/CT [5].

Retrospective institutional and population-based analyses suggest that Androgen Deprivation Therapy (ADT) with radiotherapy improves overall survival compared with ADT alone in patients with node-positive prostate cancer. These studies also consistently demonstrated that many patients with node-positive disease can achieve long-term survival and are even potentially curable [6]. In order to further optimize outcomes in this cohort, there is a need to accurately identify and delineate involved nodes in radiotherapy treatment planning.

Currently, however, there is paucity of data regarding how PSMA PET/CT modifies radiotherapeutic decisions in node positive prostate cancer treatments. This study therefore has a two-fold primary aim: (1) to map the LNs and distribution based on conventional imaging with CT compared to PSMA PET/CT and (2) determine whether the nodes identified correspond to conventional elective radiation treatment fields and if radiotherapeutic changes were made. Our secondary objective included identifying predictors for differences in PSMA PET and CT lymph node identification, assessing whether specific node locations are more likely to appear solely on PSMA PET compared to conventional CT imaging. As such, this is the first analysis to specifically examine a cohort of node-positive prostate cancer patients treated with primary radiotherapy, determining how PSMA PET/CT differs from CT in terms of node positivity, identify predictors for difference in modalities, and how this impacts radiotherapeutic decisions and treatment modifications.

## Methods

### Study design and population

This is a retrospective single-center study conducted at the Department of Radiation Oncology of Hadassah Medical Center in Jerusalem, Israel. All patients with intermediate unfavorable and high risk prostate cancer by NCCN definitions were recommended to undergo integrated modality PSMA PET/CT (with diagnostic CT). PET and contrast enhanced CT were acquired consecutively from head to the mid-thigh using a PET/CT system, approximately 60 min after the injection 68Ga-PSMA or after injection of [^18^F]PSMA-1007. This analysis focused on those patients without distant metastases but positive regional and non-regional nodes up to the aortic bifurcation found on PSMA PET/CT prior to receiving primary radiotherapy and androgen deprivation therapy between the years 2015 and 2022.

### Data collection

Patient background information was extracted from the electronic medical record including age at diagnosis, Gleason score, T stage, prostate-specific antigen (PSA) levels, and details on androgen deprivation therapy (ADT), if administered. Additionally, data on the percentage of cores involved and maximum standardized uptake value (SUV max) from the imaging reports were collected.

All reports of the PSMA PET/CT scans were reviewed and documented by a Nuclear Medicine physician. Subsequently, a dedicated radiotherapy specialist blinded to the results, reviewed first the CT without PET imaging and subsequently the fused PSMA PET/CT. Enlarged nodes on CT were identified based upon established guidelines from the consensus atlas [7]. Specifically: nodes at the aortic bifurcation, common iliac, external iliac and inguinal regions > 8 mm, nodes at internal iliac and obturator > 7 mm, nodes at mesorectal and presacral > 4 mm. PSMA PET/CT positivity was determined by uptake more than blood pool [7]. In cases of discrepancy between the report and blinded radiation oncologist an additional radiation oncologist or radiologist was requested to review.

### Radiation treatment evaluation

The patients’ radiation plans were subsequently assessed using the Varian Eclipse treatment planning software (version 16.0). The evaluation focused on determining whether there were changes from standard radiation fields used for elective nodal irradiation per consensus guidelines and whether any patients received a radiation dose boost to the LNs beyond elective nodal dosing. Changes in radiation fields and dose were correlated with the identified CT and PSMA PET/CT positive nodes compared to PSMA PET/CT only positive LNs.

### Statistical analysis

Statistical analysis was performed using IBM SPSS Statistics 29 for Windows. For the descriptive analysis, means and standard deviations, ranges, and medians were calculated. To determine the factors predicting if a patient will have LNs only in PSMA PET, chi-square and fisher tests were used for categorical variables and the t test and Mann-Whitney Test were used for numerical variables. Categorical variables evaluated included: node location, T stage, and NCCN primary risk. Numerical variables evaluated included: age, PSA value, Gleason score, SUVmax of the LNs, and percentage of cores involved. A two-sided p value of < 0.05 indicates statistical significance.

### Ethical considerations

This study received approval from the local institutional review board (IRB). The research team ensured adherence to ethical guidelines, patient confidentiality, and data protection throughout the study.

## Results

A total of 95 patients were retrospectively analyzed. Their demographic, clinical, and tumor characteristics are summarized in Table 1. The median age of the entire cohort was 72 years (range: 51–87 years). The median PSA value at diagnosis was 14.5 ng/mL (range: 1.4–630 ng/mL). The median SUVmax values of LNs was 7.05 (range: 2.2–55), The median percentage of cores involved was 83% (range: 17–100%).

**Table 1:** Demographic and tumor characteristics

Characteristic	Node presence		Total (95)
	CT and PSMA PET/CT (67)	Only PSMA PET/CT (28)	
**Age (years) (median**,** range)**	71 (51–87)	73 (58–85)	72 (51–87)
**PSA (ng/dL) (median**,** range)**	15 (1.4–630)	13.95(4-195)	14.5 (1.4–630)
**ISUP GRADE**			
1 (n,%)	1 (1)	0 (0)	1 (1)
2 + 3 (n,%)	15 (22)	7 (25)	22 (23)
4 (n,%)	11 (17)	5 (18)	16 (17)
5 (n,%)	40 (60)	16 (57)	56 (59)
**T stage**			
T1c (n,%)	5 (7)	2 (7)	7 (7)
T2a(n,%)	3 (4)	1 (4)	4 (4)
T2b (n,%)	5 (7)	2 (7)	7 (7)
T2c (n,%)	11 (16)	4 (14)	15 (16)
T3a (n,%)	11 (16)	7 (25)	18 (19)
T3b (n,%)	28 (42)	12 (43)	40 (42)
T4 (n,%)	4 (6)	0 (0)	4 (4)
**N stage**			
Regional nodes only (n,%)	43 (64)	21 (75)	64 (67)
Regional and non-regional nodes (n,%)	21 (31)	4 (14)	25 (26)
Non-regional nodes only (n,%)	3 (4)	3 (11)	6 (6)
**NCCN Primary Risk Group**			
Intermediate Unfavorable (n,%)	8 (12)	0 (0)	8 (8)
High (n,%)	15 (22)	9 (32)	24 (25)
Very high (n,%)	44 (66)	19 (68)	63 (66)
**PSMA SUV max of LN (median**,** range)**	9.45 (2.2–55)	5.35 (3-21.5)	7.05 (2.2–55)
**Percent cores involved (%) (median**,** range)**	92 (17–100)	65 (17–100)	83 (17–100)

**Fig. 1:**
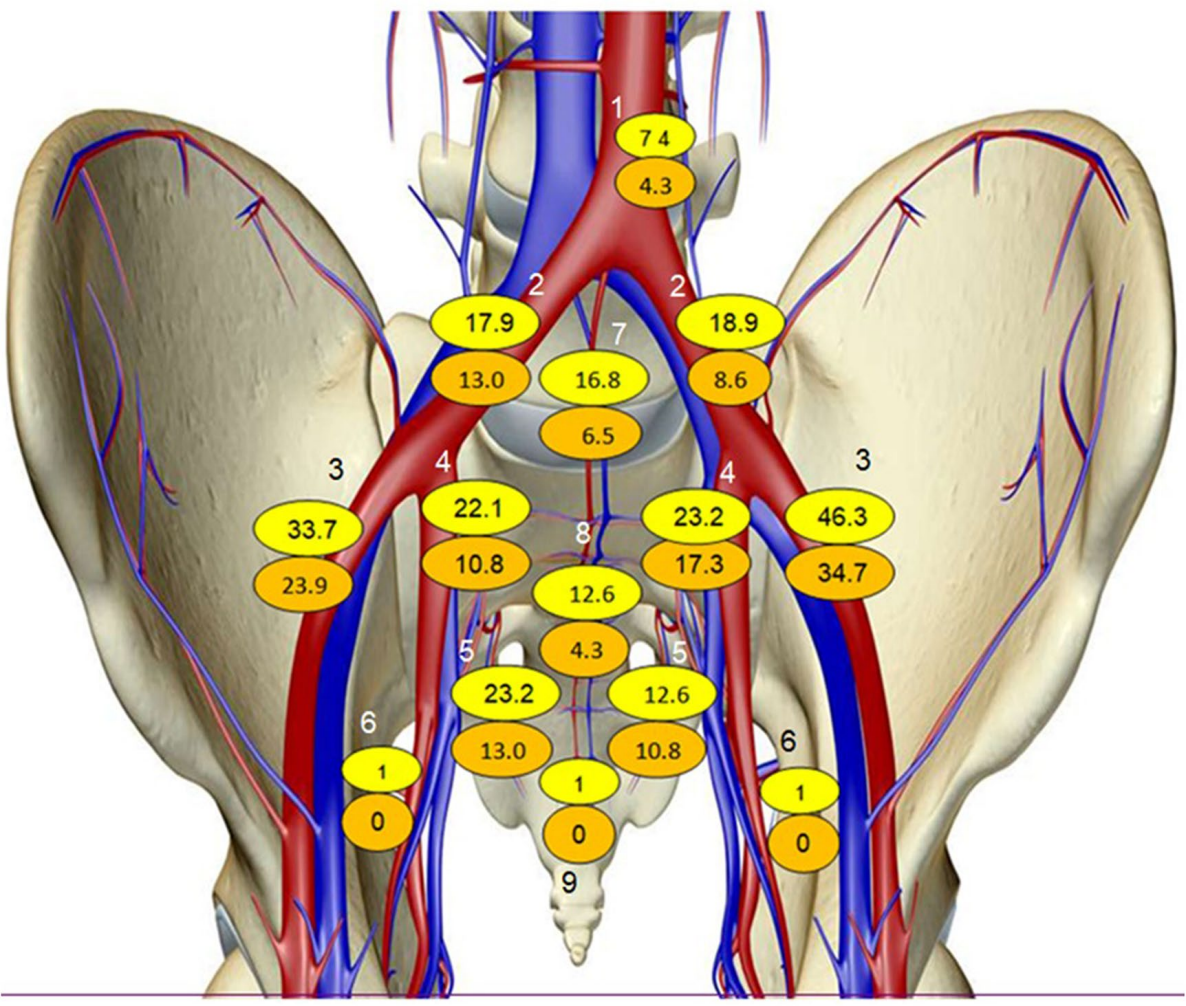
Lymph distribution (%) of all nodes (seen on both CT and PSMA PET) compared to PSMA PET only. Legend: Yellow = CT-positive/PSMA-positive nodes; Orange = CT-negative/PSMA-positive nodes 1-Aortic Bifurcation, 2-R + L Common Iliac. 3-R + L External Iliac. 4-R + L Internal Iliac. 5-R + L Obturator. 6-R + L Inguinal, 7-Presacral, 8-Mesorectal, 9-Prevesicle

A total of 226 CT and PSMA-PET-positive LN metastases were detected (Table 2; Fig. 1). The most frequent metastatic LNs were located in the external iliac (76 LNs; 34%), internal iliac (43 LNs; 19%), and common iliac (35 LNs; 15%) stations. In patients with PSMA-positive/CT-negative nodes the most common nodes were in the external iliac (27, 40%), internal iliac (13, 19%), obturator (11, 15%) stations. All the nodes on CT were detected also on PET-PSMA.

**Table 2:** Lymph node distribution based on diagnostic modality

Lymph node station	Number of nodes		
	CT-positive/PSMA-positive nodes	PSMA-positive/CT-negative nodes	Total
Aortic Bifurcation (n,%)	5 (3)	2 (3)	7 (3)
R Common iliac (n,%)	11 (7)	6 (9)	17 (8)
L Common iliac (n,%)	14 (9)	4 (6)	18 (8)
R External iliac (n,%)	21 (13)	11 (16)	32 (14)
L External iliac (n,%)	28 (18)	16 (24)	44 (19)
R Internal iliac (n,%)	16 (10)	5 (7)	21 (9)
L Internal iliac (n,%)	14 (9)	8 (12)	22 (10)
R Obturator (n,%)	16 (10)	6 (9)	22 (10)
L Obturator (n,%)	7 (4)	5 (7)	12 (5)
Presacral (n,%)	13 (8)	3 (4)	16 (7)
Mesorectal (n,%)	10 (6)	2 (3)	12 (5)
R Inguinal (n,%)	1 (1)	0 (0)	1 (0.4)
L Inguinal (n,%)	1(1)	0 (0)	1 (0.4)
Prevesical (n,%)	1 (1)	0 (0)	1 (0.4)
**Total**	**158**	**68**	**226**

**Fig. 2:**
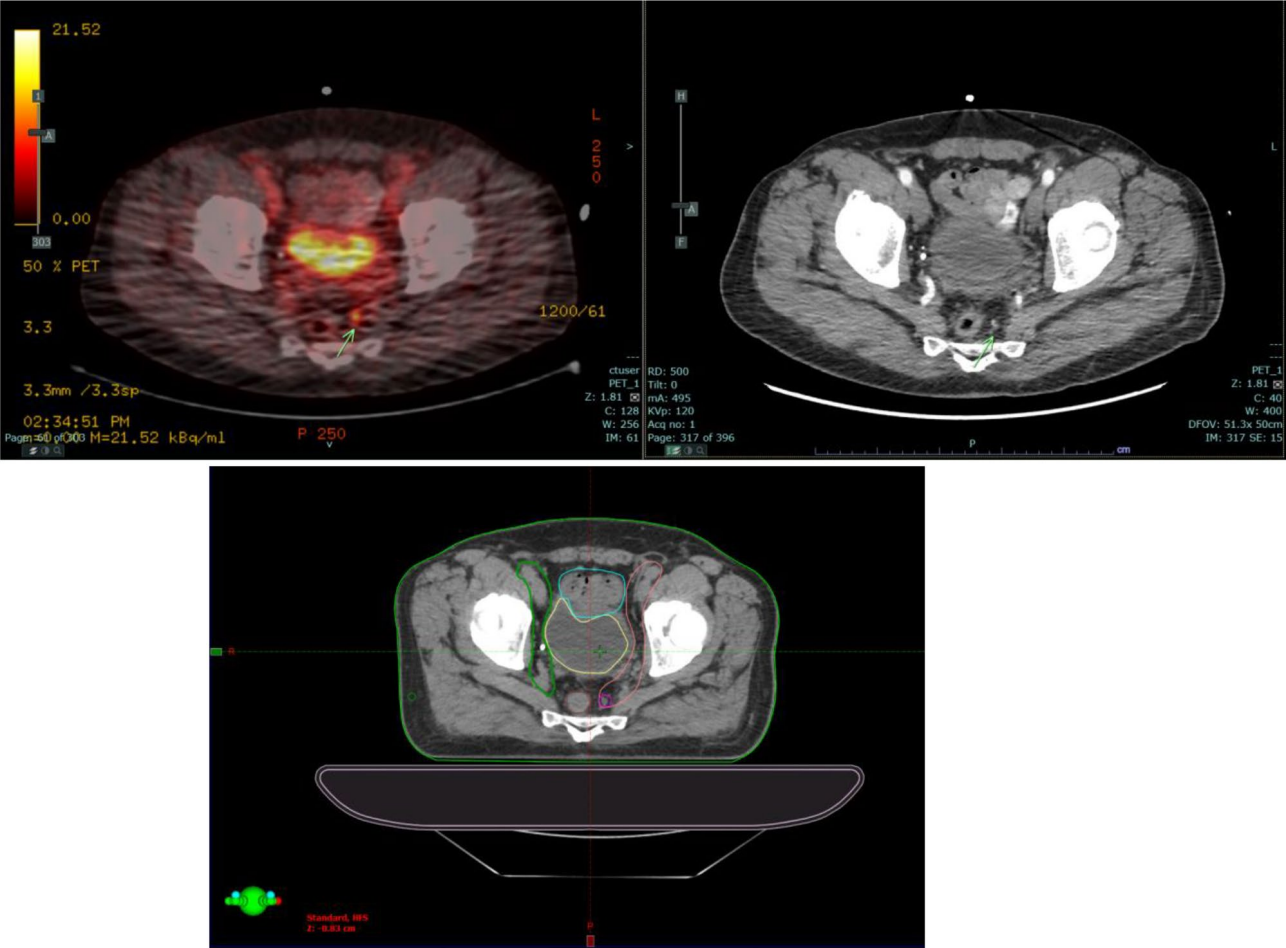
Example of PSMA PET positive mesorectal node not enlarged by CT criteria (3 mm) in which the radiotherapy contour was adjusted to cover the lymph node region when compared to the consensus atlas (Fig. 3, pictures G, H and I, Reference 7)

Among the 95 patients in the study, the PSMA PET detected additional positive nodes for 46 patients. Of these, 28 patients had nodes only detected on the PSMA PET while in 18 cases, patients initially identified with positive nodes solely through CT scans were subsequently found to harbor additional positive nodes upon thorough PSMA PET evaluation. Among all the patients in the study, changes in radiotherapy treatment fields were noted in 28 cases (29%), and 56 patients (59%) received an additional boost to the positive LN(s). Analyzing the subset of 46 patients with added nodes on PSMA PET, changes in radiotherapy treatment fields were observed in 14 cases (30%), and a dose boost was administered for 29 patients (63%). Within the subgroup of 28 patients exclusively demonstrating PSMA PET-detected nodes, changes in radiotherapy treatment fields were implemented in 5 cases (18%), and a dose boost was applied for 23 patients (83%). Radiotherapy field modifications beyond consensus elective pelvic fields included increasing the superior border of the radiotherapy field to cover nodes at the aortic bifurcation (6), coverage of mesorectal and perirectal nodes (12), or inclusion of perivesical nodes (1). See Fig. 2 for example of a mesorectal node not enlarged by CT criteria but positive on PSMA PET/CT whose radiotherapy field was adjusted to cover this node in comparison to consensus guidelines (7). When a boost to positive lymph node(s) was delivered, it was performed in the first phase of the treatment to the entire pelvis (with prostate, seminal vesicle and elective nodes) as a simultaneous integrated boost technique.

No discernible predictive factors within demographic, clinical, or tumor characteristics emerged to reliably indicate the likelihood of exclusive nodal positivity on PSMA PET scans (Table 4).

**Table 3:** Change in radiotherapy treatment design

Radiotherapy Treatment	Number of patients		
	PSMA PET/CT Additional Nodes	Nodes only detected with PSMA PET/CT	All patients
**Fields modified**			
Yes (n,%)	14 (30)	5 (18)	28 (29)
No (n,%)	32 (70)	23 (82)	67 (71)
**Dose modified**			
Yes (n,%)	29 (63)	23 (82)	56 (59)
No (n,%)	17 (37)	5 (18)	39 (41)
**Total patients**	46	28	95

**Table 4:** Clinicopathological predictors of positive nodes only on PSMA PET/CT

Parameter	*P* value
Age	0.417
PSA	0.244
ISUP GRADE	0.919
T stage	0.406
NCCN Primary Risk Group	0.104
PSMA SUV max of LN	0.132
Percent cores involved	0.193

Variables included age, PSA Value, Gleason Score, SUV Max of LN, T Stage, NCCN Primary Risk. Additionally, our investigation did not identify any statistically significant correlation between the anatomical location of specific nodes and their probability of exclusive positivity on PSMA PET imaging.

## Discussion

In this study, we conducted a comprehensive lymph node mapping using both CT and PSMA PET/CT criteria of a cohort comprising node-positive prostate cancer patients undergoing primary radiotherapy with ADT. Building upon previous research [8], our findings substantiate the superiority of PSMA PET/CT imaging, revealing additional positive LNs in 48% of cases, with 29% experiencing a consequential shift in staging from N0 to N1. Notably, our study aligns with other studies demonstrating impact of PSMA PET/CT on the N stage of prostate cancer patients (20–30%) compared to conventional imaging modalities [9, 10]. To our knowledge, this is the first analysis to specifically look at a cohort of node positive prostate cancer patients treated with primary radiotherapy and determined both how PSMA PET/CT differed from CT in terms of node positivity and how this impacted radiotherapeutic decisions and treatment modifications. By concentrating on patients with N1 status and incorporating a comparison with CT imaging, we provide a more precise portrayal of PSMA PET/CT’s influence on radiotherapeutic interventions for this subgroup, thereby contributing to the enhancement of treatment strategies.

The incidence of suspicious LNs in newly diagnosed prostate cancer patients stands at approximately 12%, and this detection rate is anticipated to rise with the increased adoption of advanced functional imaging modalities [11]. An investigation of 84 patients who underwent PSMA PET demonstrating most common lymph node locations were external and internal iliac, pelvic, common iliac, mesorectal, and presacral regions [12] which correspond to our results as well. Our exploration of the radiotherapy modifications for the 46 patients benefiting from PSMA PET revealed that alterations in radiation fields were made for 30% of cases, contrasting with the 18% observed in the subgroup where N stage was upgraded. This is consistent with other series demonstrating that PSMA PET identified disease in 16–48% of cases beyond conventional elective nodal treatment fields, as outlined in the Consensus Atlas, which has implications regarding the need to potential extend the clinical target volume in elective nodal irradiation [7, 13]. Filimonova and colleagues demonstrated that 34.7% of patients had pelvic LNs outside the NRG consensus, with perirectal and common iliac nodes being the most common nodes [14]. Our study is in line with this finding with perirectal and inclusion of nodes up to the bifurcation were the most common reason for radiotherapeutic field modification in comparison to elective fields. It has been our practice in patients with non-regional nodes up to the aortic bifurcation to treat with primary radiotherapy and ADT. While these nodes are considered M1a disease based on the molecular imaging TNM system, retrospective data has demonstrated similar outcomes when treated with curative whole pelvic RT and long-term ADT compared to those with regional nodes [15] supporting this approach. This underscores the evolving landscape in prostate cancer management, urging clinicians to consider the broader disease extent revealed by PSMA PET/CT and adjust treatment strategies accordingly.

Dose modifications were more prevalent, occurring in 63% of the total group and, notably, in 82% of patients with an upgraded N stage with PSMA PET/CT. This underscores a more significant impact on treatment planning for patients transitioning from N0 to N1, who would otherwise receive a lower elective dose of radiation to the whole pelvis or no radiotherapy to the pelvis at all depending on institutional preferences. However, despite the notable impact of PSMA PET/CT on radiotherapeutic boosts, there is minimal data regarding accurate guidelines for radiation boost in prostate cancer-positive pelvic LN. While most studies on PET-guided dose-painting have focused on 18 F-fluorodeoxyglucose (FDG) or hypoxia PET tracers, concerns about their utility for dose-painting persist. In contrast, PSMA ligands exhibit promise for radiotherapeutic dose-escalation due to their relatively high correlation with histopathological findings [16]. This leads us to advocate for further exploration of the impact of SUVmax in pet-positive LN on the radiation dosage administered as a boost to the treatment.

Despite the valuable insights gained from our study, several limitations warrant acknowledgment. These include the retrospective nature of our analysis, potential selection bias, and the need for larger, prospective cohorts to validate our findings. Additionally, as not all of the imaging was performed at the same institution, the generalizability of our results may be limited by variations in institutional protocols and imaging technologies. Potentially, reactive nodes may be treated as a false positive with low grade activity. However, we attempted to mitigate this issue by taking a cohort who were treated under a multidisciplinary team and thus had a high clinical suspicion of having node positive prostate cancer in addition to their imaging findings. Moreover, we were unable to identify any predictors among patient demographics, clinical parameters, tumor characteristics, or lymph node locations that could explain the observed disparities between CT and PSMA PET imaging. Lastly, to establish the clinical impact of radiation modifications in light of PSMA PET/CT findings, further studies are warranted. In our study, many patients had a follow-up period of less than two years while on ADT, making the analysis of oncologic outcomes not clinically meaningful.

In conclusion, our findings advocate for the routine incorporation of PSMA PET/CT into the diagnostic staging for node-positive prostate cancer, acknowledging its pivotal role in shaping personalized and effective radiotherapy strategies. As the field continues to evolve, collaboration across institutions and concerted efforts in research will further elucidate the multifaceted impact of PSMA PET, ultimately enhancing the precision of treatment decisions and improving outcomes for patients with node-positive prostate cancer.

## Data Availability

No datasets were generated or analysed during the current study.
